# Saccharomyces cerevisiae isolated in a degloving baker’s machine injury, a contamination or infection – a case report

**DOI:** 10.1186/s12879-026-12764-2

**Published:** 2026-02-14

**Authors:** Heloi Stefani, Daniel Hanawa Morisaki, Boanerge Ojeda Ch., Madhu Babu Adusumilli, Angel Lorenzo Porras, Maha Albukhari, Folusakin Ayoade

**Affiliations:** 1 Department of Internal Medicine, Jacobi Medical Center - Albert Einstein College of Medicine, New York, USA; 2 Universidad Peruana Cayetano Heredia, San Martin de Porres, Peru; 3 Universisidad Nacional Autonoma de Nicaragua, Managua, Nicaragua; 4 All Indian Institute of Medical Sciences (AIIMS), Bhopal, India; 5 Baptist Health Systems, Miami, USA; 6 Division of Infectious Diseases, University of Miami Miller School of Medicine, Miami, USA

**Keywords:** *Saccharomyces cerevisiae*, Baker, Degloving Laceration, Wound contaminant, Cellulites

## Abstract

Deep tissue and bloodstream yeast infections are associated with poor outcomes due to rapid progression, antifungal resistance, and immune evasion. Differentiating between wound contamination and true fungal infection remains a clinical challenge, with limited guidance on the use of prophylactic antifungal therapy in this setting. This article presents a rare case of an immunocompetent patient who sustained a degloving hand injury and radius fracture from a bread kneading machine, with subsequent deep tissue cultures growing *Saccharomyces cerevisiae*. The case underscores the potential risk of fungal infection in occupational injuries involving dough and emphasizes the importance of early identification and consideration of prophylactic antifungal treatment in similar scenarios.

## Introduction

*Saccharomyces cerevisiae*, a yeast belonging to the Ascomycetes division and the *Saccharomyces* class, is commonly found in the human gastrointestinal and respiratory tracts. It also exists in plants and soil and is widely recognized as baker’s or brewer’s yeast due to its extensive use in baking and brewing processes [1].

Previously considered non-pathogenic, *S. cerevisiae* has been utilized as a probiotic to address antibiotic-related diarrhea and promote the healing of chronic wounds [2]. Nevertheless, this yeast can lead to severe infections in immunocompromised patients, particularly those undergoing immunosuppressive therapy, broad-spectrum antibiotics, chemotherapy, or intravascular catheters [3]. Furthermore, it has been reported to impact immunocompetent hosts, contributing to conditions such as vaginitis and leading to fungemia and osteomyelitis in patients with traumatic injuries [4, 5].

In terms of susceptibility, *S. cerevisiae* has demonstrated variable resistance to antifungal agents like fluconazole and itraconazole while being sensitive to voriconazole, caspofungin, amphotericin, and flucytosine [1, 6, 7]. This resistance primarily arises from the upregulation of the ERG genes and recombinant efflux pumps, a mechanism it shares with various Candida serotypes, especially the challenging *Candida auris* [7, 8].

In situations where the barriers of the innate immune system are compromised, allowing microorganisms to breach the skin, muscular fascia, and periosteum, as observed in this case report, it is crucial to give due consideration to antifungal prophylaxis. This is especially important to mitigate or prevent potential complications, such as cellulitis, osteomyelitis, fungemia, and other disseminated infections that are possible with this fungus [3, 5].

## Case report

**Fig. 1:**
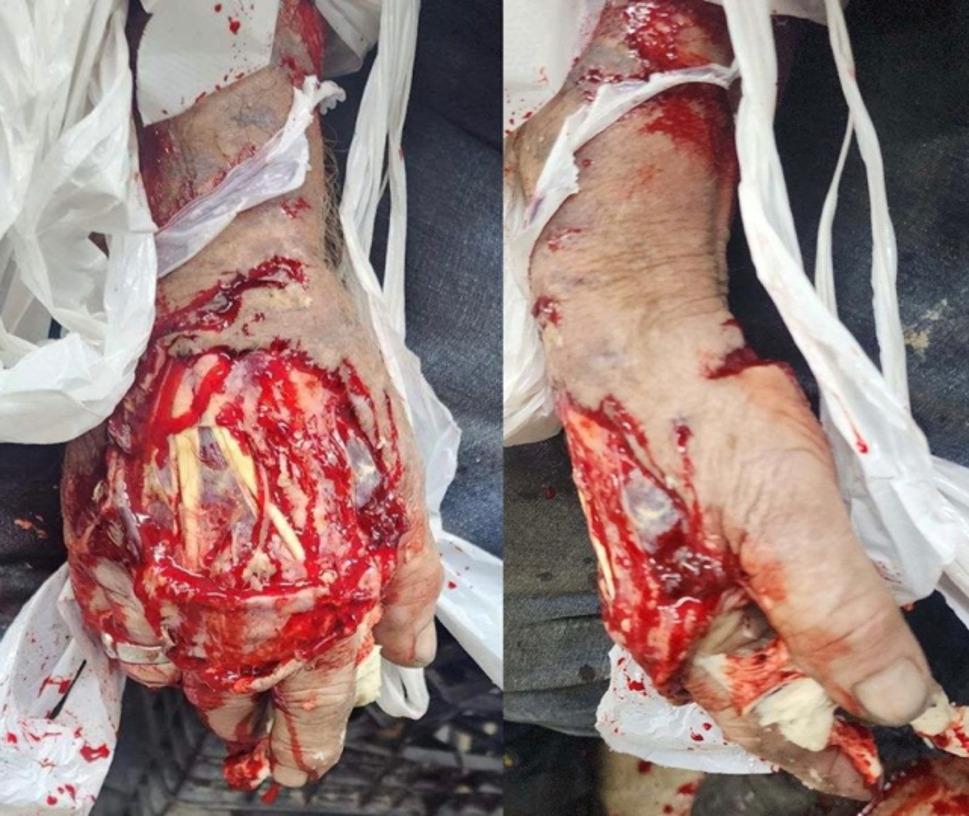
Clinical Image. Traumatic right-hand degloving lauceratyion injury

Our case involves a 75-year-old male baker with a previous medical history of hypertension, hyperlipidemia, and coronary artery disease. He presented at the emergency department after sustaining a degloving crush injury aggravated by thermal damage to his right hand and a unicortical fracture across the radial shaft while operating a bread kneading machine (Fig. 1), (day 0).

**Fig. 2:**
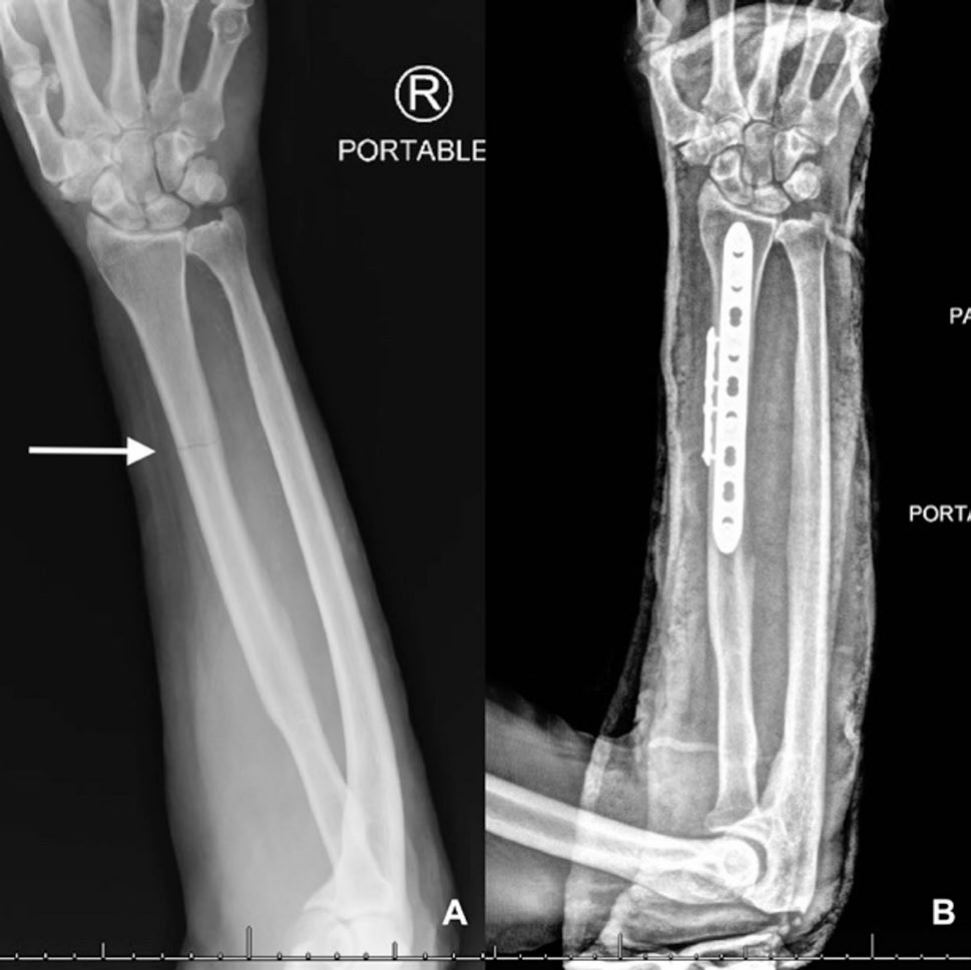
Radiography (Rx). **A**- Bone radiograph non-displaced fracture of the right radial diaphysis. **B**- Interval plate and screw fixation of the radial diaphysis fracture, overlying cast

Initial radiograph imaging disclosed an acute, non-displaced fracture of the right radial diaphysis (Fig. 2a). Subsequently, the patient underwent a surgical intervention involving copious irrigation, debridement of necrotic tissue, and application of wound vacuum therapy. Tissue biopsy and bacterial, fungal, and mycobacterial cultures were obtained, and the patient was commenced on Ceftriaxone 2 g intravenously daily.

**Fig. 3:**
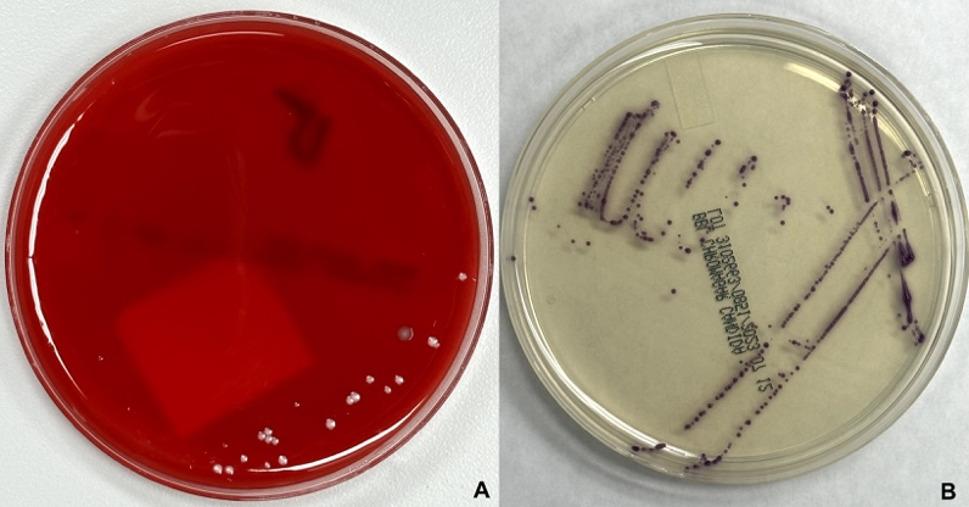
Mycological cultures of deep wound cultures at debridement. **A**- Small (2–3 mm diameter), round, smooth, pink with white edge colonies of *Saccharomyces cerevisiae* in blood agar. **B**- Small (1–2 mm diameter), round, smooth, purple edge colonies *Saccharomyces cerevisiae* in CHROMagar-Candida

On the second day after admission, laboratory results showed normal complete blood count, hemoglobin, renal, and liver function tests. Mycological culture of the deep wound after 48 h revealed the presence of small, 2–3 mm, round, smooth, pink colonies in blood agar (Fig. 3A). Additionally, CHROMagar Candida displayed compact 1–2 mm round colonies, exhibiting a smooth purple appearance (Fig. 3B), suggestive of yeast growth. The yeast’s identification was achieved through direct microscopy and matrix-assisted laser desorption/ionization time-of-flight mass spectrometry (MALDI-TOF MS) analysis of the mycological cultures. Consequently, the patient was taken to the operating room, where he underwent a secondary surgical revision, further debridement, an open reduction procedure, and internal fixation of the right radius [Figure 2b].

Given concern for high risk of secondary infection in this grossly contaminated wound, the patient was started on an empirical oral regimen involving trimethoprim-sulfamethoxazole 800/160 mg twice daily for five days, alongside fluconazole 500 mg daily (weight-based dosing) for six weeks. Mycological susceptibilities were requested, and the patient was discharged and provided with outpatient follow-up appointments.

Nine days after the initial injury, antifungal susceptibility results became available, revealing minimum inhibitory concentration (MIC) values as follows: amphotericin B 1 mcg/ml, fluconazole 4 mcg/ml, and voriconazole 0.06 mcg/ml (no definitive breakpoints established yet for *S. cerevisiae*). Following the review of the susceptibility antifungal results, fluconazole was discontinued, and the patient was started on an oral regimen consisting of voriconazole 400 mg two times a day on day one, and subsequently 300 mg two times a day for three months.

Follow-up visits were consistently upheld, ensuring that the voriconazole’s therapeutic range remained within 1–5 µg/ml, which was mostly achieved except for one occasion when levels were subtherapeutic. Except for mild facial and leg edema, the antifungal therapy was well tolerated without any evidence of long-term adverse effects. Wound care management continued with the use of wet-to-dry dressings while on antifungal therapy.

**Fig. 4:**
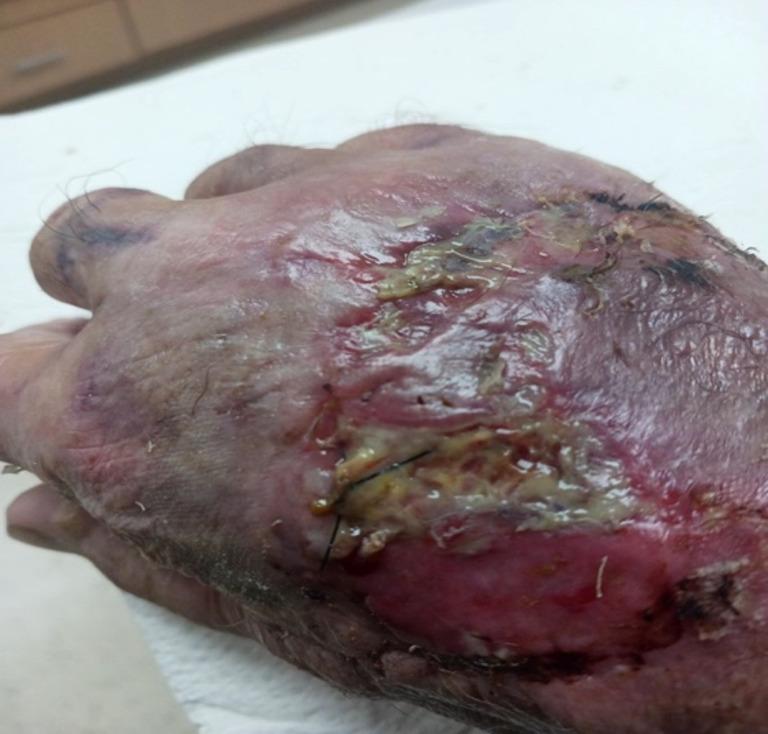
Clinical Image follow-up day 64. Chronic wound site infection: the dorsal region displayed areas of denuded epidermis, exudative fibrinoid thick secretions around the incision site, and edema on the right hand, with concern for early cellulitis

Sixty-four days following the initial injury, the patient exhibited clinical deterioration of his wound. Notably, the dorsal region displayed areas of denuded epidermis, accompanied by exudative fibrinoid thick secretions involving the incision site and edema of the right hand (Fig. 4). This overall picture suggests a wound and soft tissue infection and possible cellulitis.

**Fig. 5:**
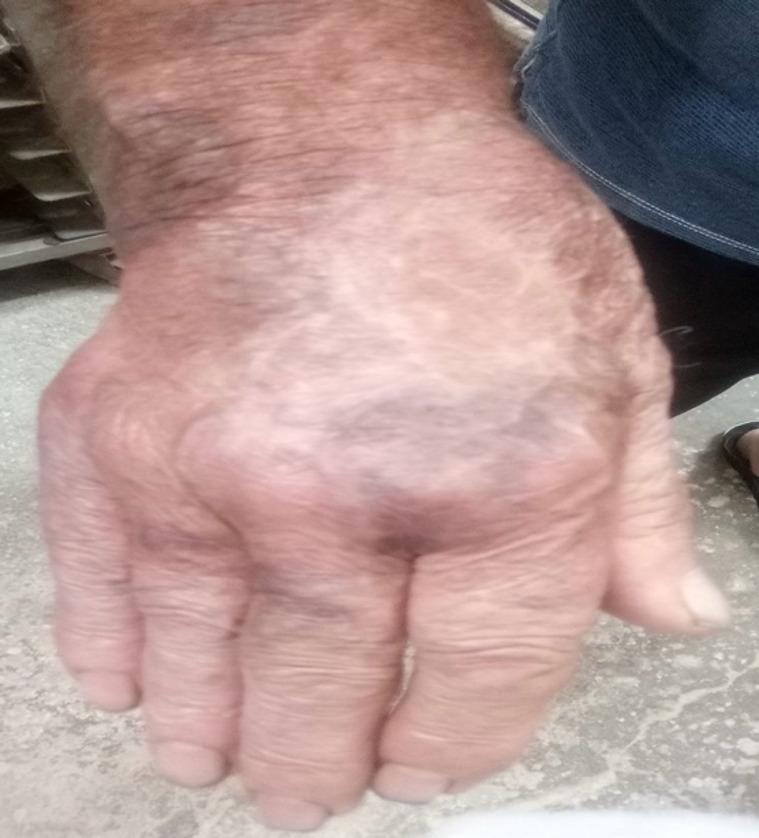
Clinical Image follow-up, day 180, right hand showing complete wound healing with some scarring

The patient completed a 90-day course of voriconazole, with no additional antibiotics given, resulting in complete regression of the inflammatory process. The wound fully healed, with the patient reporting only a minimal area of residual skin induration. Overall hand function was normal, and the patient was able to return to work (Fig. 5).

## Discussion

*S. cerevisiae* has been recognized as a potential pathogen in both monomicrobial and polymicrobial infections following deep tissue trauma, often leading to bone and joint complications. These cases pose a diagnostic challenge, particularly when the organism is isolated from traumatic wounds, making it difficult to distinguish true infection from contamination. Decisions regarding the initiation of prophylactic antibiotic or antifungal therapy postoperatively remain complex due to the rarity of such cases and the lack of established guidelines.

Although complicated wound infections caused by *S. cerevisiae* are uncommon, they can occur even in immunocompetent individuals, as demonstrated in our case. This organism should be considered a potential pathogen, particularly in individuals occupationally exposed to baker’s or brewer’s yeast. Even with minimal clinical suspicion, differentiating between colonization and true infection remains challenging [9, 10].

Wound healing is a dynamic process involving hemostasis, inflammation, proliferation, and remodeling phases, with the ultimate goal of restoring tissue integrity. Chronic or complicated wounds—those persisting for more than three months—are frequently associated with poor vascularization, infection, or systemic comorbidities. Wound infections can present with local signs such as erythema, edema, warmth, purulent discharge, or increased pain. In patients with multiple comorbidities, more subtle manifestations such as delayed healing or systemic symptoms may predominate, necessitating a thorough clinical assessment and exclusion of other infection sources [11].

The early recognition of infection is therefore critical, as the mere presence of a microorganism in a wound does not confirm infection; it may instead indicate contamination. Differentiating between the two is essential, as it significantly influences the choice and intensity of clinical management strategies [10, 12].

While *S. cerevisiae* generally remains susceptible to echinocandins, amphotericin B, and flucytosine, many reports suggest in vitro resistance to azoles, particularly fluconazole [1, 6, 7]. Susceptibility to voriconazole, as reported in some studies, is, however, generally high, with rates above 90% [13, 14]. One of the primary mechanisms of resistance involves alterations in the drug’s binding target, either through genetic modification or overexpression. Similar to *Candida albicans*, *S. cerevisiae* shares resistance mechanisms, including mutations in the ERG11 gene, which confer resistance to azoles and polyenes. Echinocandin resistance is primarily mediated through mutations in FKS1, while efflux pump overexpression also contributes to azole resistance by reducing intracellular drug concentrations [8].

Despite early prophylactic treatment, our patient developed signs of wound infection and concern for early cellulitis. This, however, improved with the continuation of antifungal therapy and wound care. It is conceivable that the patient could have fared worse if antifungal therapy had not been initiated after *S. cerevisiae* was confirmed from tissue culture.

Cutaneous and soft tissue infections (SSTI) caused by *S. cerevisiae* are exceptionally rare, despite the organism’s ubiquitous use in food and probiotics. One of the earliest reports came from Wilson et al. [15], who described an infection in the vulva in a bread-making woman with apparent transmission to her partner, underscoring the potential for mucocutaneous colonization in otherwise healthy individuals. A decade later, Almanza et al. [16] reported a fulminant case of post-traumatic cellulitis developing within four hours in a wine cellar worker, where occupational exposure to grape-associated *S. cerevisiae* was implicated as the etiologic agent. Subsequently, Lee et al. [17] documented dermal abscesses in a steroid-treated patient that improved with fluconazole therapy, while Williams et al. [18] described cutaneous septic emboli in an immunocompromised patient with relapsed acute myeloid leukemia, representing secondary skin involvement from fungemia. More recently, Belmourida et al. [19] reported the first primary diffuse maculopapular infection in an immunocompetent patient, highlighting that clinically significant disease is not confined to immunosuppressed hosts.

*S. cerevisiae* is generally susceptible to echinocandins, amphotericin B, and flucytosine; however, in vitro resistance to azoles, particularly fluconazole, has been frequently reported, largely due to mechanisms shared with *Candida albicans*, including ERG11 mutations, alterations or overexpression of drug targets, FKS1 mutations conferring echinocandin resistance, and efflux pump overexpression leading to reduced intracellular azole concentrations [1, 6–8]. In our case, the isolate remained susceptible to voriconazole. Although antifungal susceptibility testing was not routinely performed in previously reported cases, larger series demonstrate consistent activity of amphotericin B and flucytosine, variable susceptibility to fluconazole, and overall good activity of echinocandins [18, 19]. Clinical outcomes have been heterogeneous, with successful azole therapy reported in at least one case, while others were limited by incomplete follow-up or disseminated infection [15–17]. Collectively, these findings highlight that although *S. cerevisiae* skin and soft tissue infections are rare, they should be considered in the differential diagnosis of atypical or treatment-refractory skin infections when common pathogens are not identified; previously reported cases in the English literature are summarized in Table 1.

**Table 1:** Published cases of Saccharomyces cerevisiae skin and soft tissue infections

Manuscript reference	Year	Age/ Gender	Host factors	Infection type	Microbiology	Antifungal susceptibilities	Treatment
Wilson et al. [15]	1987	30 F	Immunocompetent, history of bread baking	Vulval infection, with whitish vaginal discharge	Vaginal discharge cultures grew *S. cerevisiae.* Test for *Candida albicans* was negative	Not reported	Fluconazole 50 mg daily for 3 days
Lee et al. [15]	1997	62 F	Immunosuppressed (Long-term systemic steroids)	Left thigh deep dermal abscess/nodules	Culture of nodular lesion biopsy grew Saccharomyces spp.	Not reported	Fluconazole 150 mg/day ×14 days
Williams et al. [16]	2007	51 F	Immunosuppressed (Relapsed AML M6 subtype, Allogeneic Bone Marrow transplant x 2; Hickman line)	Cutaneous septic emboli and Hickman line infection	Skin biopsy and Hickman line grew *S. cerevisiae*	Not reported	Not detailed
Belmourida et al. [17]	2021	72 F	Immunocompetent	Diffuse chronic maculopapular skin lesions	Cultures of the biopsy of two lesions grew *S. cerevisiae*	Not reported	None (patient left against medical advice)

Ultimately, after completing a 90-day course of voriconazole, the patient achieved complete wound healing. He reported only minimal residual skin tightness, with full restoration of hand function and no limitations in daily activities or work.

A key limitation in this case was the initial choice of fluconazole for initial prophylaxis, despite known resistance patterns in some published reports. We chose fluconazole initially, given the evidence of a few reports showing good response to this antifungal [13, 15], the excellent tolerability, and the fact that this fungus isolation represented contamination rather than infection early on. While fluconazole is more convenient and better tolerated, the use of voriconazole from the outset might have been more appropriate given the potential for invasive fungal disease and the general poor susceptibilities reported to fluconazole for *S. cerevisiae* [18–20]. However, this decision remains debatable, especially in the absence of confirmed infection and considering voriconazole’s need for therapeutic drug monitoring and potential side effects.

In conclusion, we believe that significant fungal deep wound contamination or infection with *S. cerevisiae* warrants prompt and aggressive treatment, beginning with empirical antifungal therapy—preferably voriconazole or an echinocandin—with subsequent antimicrobial adjustment based on fungal susceptibility results.

## Data Availability

The datasets used and/or analyzed during the current study are available from the corresponding author on reasonable request.

## Abbreviations

ERG genes: Ergosterol biosynthesis genes
MALDI: TOF MS–Matrix–Assisted Laser Desorption/Ionization Time–of–Flight Mass Spectrometry
MIC: Minimum Inhibitory Concentration
SSTI: Skin and Soft Tissue Infection
AML: Acute Myeloid Leukemia

## References

[1] Muñoz P, Bouza E, Cuenca-Estrella M, et al. Saccharomyces cerevisiae fungemia: an emerging infectious disease. Clin Infect Dis. 2005;40(11):1625–34. 10.1086/429916. Epub 2005 Apr 25. PMID: 15889360. 15889360 10.1086/429916

[2] Gupta P, Singh YP, Taneja A. A friend or foe in ICU (A case report with Solution). Indian J Crit Care Med. 2019;23(9):430–1. 10.5005/jp-journals-10071-23239. PMID: 31645830; PMCID: PMC6775723. Saccharomyces. 31645830 10.5005/jp-journals-10071-23239 PMC6775723

[3] Aucott JN, Fayen J, Grossnicklas H, Morrissey A, Lederman MM, Salata RA. Invasive infection with Saccharomyces cerevisiae: report of three cases and review. Rev Infect Dis. 1990 May-Jun;12(3):406 – 11. 10.1093/clinids/12.3.406. PMID: 2193348. 10.1093/clinids/12.3.406 2193348

[4] Papaemmanouil V, Georgogiannis N, Plega M, et al. Prevalence and susceptibility of Saccharomyces cerevisiae causing vaginitis in Greek women. Anaerobe. 2011;17(6):298–9. 10.1016/j.anaerobe.2011.04.008. Epub 2011 Apr 29. PMID: 21549212. 21549212 10.1016/j.anaerobe.2011.04.008

[5] Seng P, Cerlier A, Cassagne C, Coulange M, Legré R, Stein A. Saccharomyces cerevisiae osteomyelitis in an immunocompetent Baker. IDCases. 2016;5:1–3. 10.1016/j.idcr.2016.05.002. PMID: 27347482; PMCID: PMC4909721. 27347482 10.1016/j.idcr.2016.05.002 PMC4909721

[6] Pérez-Cantero A, Thomson P, Paredes K, Guarro J, Capilla J. Antifungal susceptibility of Saccharomyces cerevisiae and therapy in a murine model of disseminated infection. Rev Iberoam Micol. 2019 Jan-Mar;36(1):37–40. doi: 10.1016/j.riam.2018.04.004. Epub 2019 Feb 11. PMID: 30765275. 10.1016/j.riam.2018.04.004 30765275

[7] Toepfer S, Lackner M, Keniya MV, Monk BC. Functional expression of Recombinant *Candida auris* proteins in *Saccharomyces cerevisiae* enables Azole susceptibility evaluation and drug discovery. J Fungi (Basel). 2023;9(2):168. 10.3390/jof9020168. PMID: 36836283; PMCID: PMC9960696. 36836283 10.3390/jof9020168 PMC9960696

[8] Lee Y, Robbins N, Cowen LE. Molecular mechanisms governing antifungal drug resistance. NPJ Antimicrob Resist. 2023;1(1):5. 10.1038/s44259-023-00007-2. Epub 2023 Jul 17. PMID: 38686214; PMCID: PMC11057204. 38686214 10.1038/s44259-023-00007-2 PMC11057204

[9] Souza Goebel C, de Mattos Oliveira F, Severo LC. Infección por Saccharomyces cerevisiae [Saccharomyces cerevisiae infections]. Rev Iberoam Micol. 2013 Jul-Sep;30(3):205-8. Spanish. 10.1016/j.riam.2013.03.001. Epub 2013 Apr 11. PMID: 23583718. 10.1016/j.riam.2013.03.001 23583718

[10] Ganesan A, Shaikh F, Bradley W, Tribble DR, Infectious Disease Clinical Research Program Trauma Infectious Disease Outcomes Study Group, et al. Classification of Trauma-Associated invasive fungal infections to support wound treatment decisions. Emerg Infect Dis. 2019;25(9):1639–47. 10.3201/eid2509.190168. PMID: 31441428; PMCID: PMC6711217. 31441428 10.3201/eid2509.190168 PMC6711217

[11] Carville K, Cuddigan J, Fletcher J, et al. Wound infection in clinical practice. Int Wound J. 2008;5(s3):iii–11. 10.1111/j.1742-481x.2008.00488.x. 10.1111/j.1742-481X.2008.00488.x PMC7951552 18489408

[12] Metsemakers WJ, Morgenstern M, McNally MA, et al. Fracture-related infection: A consensus on definition from an international expert group. Injury. 2018;49(3):505–10. 10.1016/j.injury.2017.08.040. Epub 2017 Aug 24. PMID: 28867644. 28867644 10.1016/j.injury.2017.08.040

[13] Górzyńska A, Kondracka K, Korzeniowska-Kowal A, Nawrot U. Antifungal susceptibility of *Saccharomyces cerevisiae* isolated from clinical specimens. Pathogens. 2024;13(3):248. 38535591 10.3390/pathogens13030248 PMC10974509

[14] Yan Z, Fu Y, Tan X, et al. Isolate distribution and antifungal susceptibility of *Saccharomyces cerevisiae* in the National regional medical center of Southwest China for women and children during 2018–2023. BMC Microbiol. 2024;24:345. 39271978 10.1186/s12866-024-03506-y PMC11401246

[15] Wilson JD, Jones BM, Kinghorn GR. Bread-making as a source of vaginal infection with *Saccharomyces cerevisiae*: report of a case in a woman and apparent transmission to her partner. Sex Transm Dis. 1988;15(1):35–6. 3282334 10.1097/00007435-198801000-00008

[16] Almanza L, Debien B, Fontaine B, Brinquin L. Quatre heures pour Un record, Ou Une cellulite vraiment foudroyante: Saccharomyces cerevisiae peut-elle En être La cause? Ann Fr Anesth Reanim. 1998;17(1):130–2. 9750709 10.1016/s0750-7658(98)80061-0

[17] Lee HK, et al. A case of opportunistic skin infection with Saccharomyces. Ann Dermatol. 1997;9(1):41–5.

[18] Williams JS, et al. *Saccharomyces cerevisiae* emboli in an immunocompromised patient. Clin Exp Dermatol. 2007;32(4):395–7. 17376213 10.1111/j.1365-2230.2007.02375.x

[19] Belmourida S, et al. Skin infection with *Saccharomyces cerevisiae* in an immunocompetent patient: an exceptional infection. Our Dermatol Online. 2021;12(4):466–7.

[20] Enache-Angoulvant A, Hennequin C. Invasive *Saccharomyces* infection: a comprehensive review. Clin Infect Dis. 2005;41(11):1559–68. 16267727 10.1086/497832

